# The Antiviral Activity of Polyphenols

**DOI:** 10.1002/mnfr.70042

**Published:** 2025-04-01

**Authors:** Markus Burkard, Alban Piotrowsky, Christian Leischner, Katja Detert, Sascha Venturelli, Luigi Marongiu

**Affiliations:** ^1^ Department of Nutritional Biochemistry University of Hohenheim Stuttgart Germany; ^2^ Department of Vegetative and Clinical Physiology Institute of Physiology University of Tuebingen Tuebingen Germany

**Keywords:** antivirals, enveloped virus, polyphenols

## Abstract

Polyphenols are secondary metabolites produced by a large variety of plants. These compounds that comprise the class of phenolic acids, stilbenes, lignans, coumarins, flavonoids, and tannins have a wide range of employment, from food production to medical usages. Among the beneficial applications of polyphenols, their antiviral activity is gaining importance due to the increased prevalence of drug‐resistant viruses such as herpes and hepatitis B viruses. In the present review, we provide an overview of the most promising or commonly used antiviral polyphenols and their mechanisms of action focusing on their effects on enveloped viruses of clinical importance (double‐stranded linear or partially double‐stranded circular DNA viruses, negative sense single‐stranded RNA viruses with nonsegmented or segmented genomes, and positive sense single‐stranded RNA viruses). The present work emphasizes the relevance of polyphenols, in particular epigallocatechin‐3‐gallate and resveratrol, as alternative or supportive antivirals. Polyphenols could interfere with virtually all steps of viral infection, from the adsorption to the release of viral particles. The activity of polyphenols against viruses is especially relevant given the risk of widespread outbreaks associated with viruses, remarked by the recent COVID‐19 pandemic.

## Graphical abstract

Polyphenols demonstrate remarkable antiviral properties by effectively disrupting multiple biochemical processes essential for viral replication.

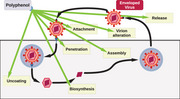

**Abbreviations**
ACE2: angiotensin‐converting enzyme 2
AMR: antimicrobial‐resistant microbes
BZLF1: BamHI Z fragment leftward open reading Frame 1
CBH: chronic B hepatitis
CC_50_
: 50% cytotoxic concentration
CDK‐9: cyclin‐dependent kinase 9
CHIKV: chikungunya virus
DENV: dengue virus
EBOV: Ebola virus
EC_50_
: 50% effective concentration
EGCG: epigallocatechin‐3‐gallate
FLUV: influenza virus
FXR: farnesoid‐X‐receptor
GI: gastrointestinal
HA: hemagglutinin
HBcAg
: HBV core antigen
HBeAg: HBV extracellular antigen
HBsAg: HBV surface antigen
HBV: hepatitis B virus
HBx: hepatitis B viral protein X
HCoV: human coronavirus
HCV: hepatitis C virus
HHV: human herpesvirus
HO‐1: heme oxygenase‐1
HVEM: herpesvirus entry mediator
IC_50_
: 50% inhibitory concentration
ICP22: infected‐cell protein 22
IE: immediate‐early
IFN‐I: type one interferon
IRF7: interferon regulatory Factor 7
ISG15: interferon‐stimulated gene 15
JEV: Japanese encephalitis virus
LMP‐1: latent membrane protein 1
MERS‐CoV: Middle East respiratory syndrome coronavirus
MeV: measles virus
M^pro^
: main protease
NA: neuraminidase
Neu5Ac: *N*‐acetylneuraminic acid
NF‐κB: nuclear factor kappa B
NS3: nonstructural protein 3
NTCP: Na^+^/taurocholate cotransporter polypeptide
NTPase: nucleoside‐triphosphatase
OAS3: 2′‐5′‐oligoadenylate synthetase 3
pg: pre‐genomic
PGG: pentagalloylglucose
PKR: protein kinase RNA‐activated
PL^pro^
: papain‐like protease
RdRp: RNA‐dependent RNA‐polymerase
RNaseH: ribonuclease H
RpII: RNA polymerase II
RSV: respiratory syncytial virus
Rta: replication and transcription activation
RVFV: Rift Valley fever virus
SARS‐CoV: severe acute respiratory syndrome coronavirus
SI: selectivity index
SLAM: signaling lymphocytic activation molecule
STAT1: signal transducer and activator of transcription 1
TCQA: 3,4,5‐tri‐*O*‐caffeoylquinic acid
TFIID: transcription Factor II D
TGG: 1,2,3,6‐tetragalloyl glucose
TI: therapeutic index
TLR3: toll‐like receptor 3
VACV: vaccinia virus
VP16: viral protein 16
VSV: vesicular stomatitis Indiana virus
WNV: West Nile virus
XN: xanthohumol
ZIKV: Zika virus

## 1 Introduction

**FIGURE 1:**
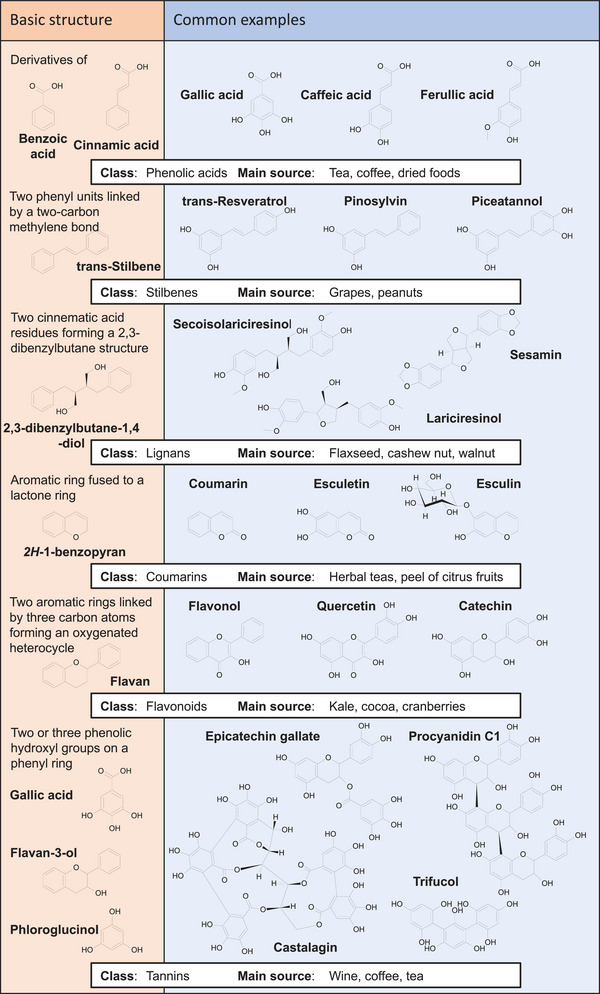
Overview of polyphenols. The basic structure of polyphenols (left column) with some of the most common examples for each class (right column). The main dietary sources for the represented polyphenols are provided in the boxes underneath the structures.

Polyphenols are secondary metabolites produced by a wide range of plants that have been employed for centuries for their beneficial properties in popular medicine [1]. In addition, polyphenols are used to prepare food and beverages to maintain the organoleptic properties of the preparations, making these molecules particularly suitable for the food processing market [2]. Polyphenols are derivatives of phenol (or benzenol), which is an aromatic compound formed by phenyl (⌬) and hydroxyl (−OH) groups. More than 8000 polyphenols have been described so far, subdivided into six classes (Figure 1 and Table 1) [3]. A particularly relevant class of polyphenols is that of flavonoids, derivatives of the flavan ring [4]. In recent times, the properties of polyphenols have been investigated scientifically to apply them for applications ranging from immune modulation to antioxidant treatment and blood pressure management [5]. Moreover, polyphenols are attractive for their anti‐bacterial properties due to the need for antibiotic alternatives following the increased spreading of antimicrobial‐resistant microbes (AMR) [6]. Polyphenols also show antiviral activities.

**TABLE 1:** Classes of polyphenols mentioned in the present review.

Class	Characteristics	Main dietary sources	Ref.
Phenolic acids	Derivates of benzoic acid or cinnamic acid	Tea, coffee, dried fruits	[170]
Stilbenes	Two phenyl units linked by a two‐carbon methylene bond	Grapes, peanuts	[171]
Lignans	Two cinnamic acid residues forming a 2,3‐dibenzylbutane structure	Flaxseed, cashew nut, walnut	[171, 172]
Coumarins	Aromatic ring fused to a lactone ring	Herbal teas, peel of citrus fruits	[173]
Flavonoids	Two aromatic rings linked by three carbon atoms forming an oxygenated heterocycle	Citrus fruits, chocolate, berries	[174]
Tannins	Two or three phenolic hydroxyl groups on a phenyl ring	Wine, coffee, tea	[175]

Viruses are obligate endoparasitic microorganisms composed of a genome encapsulated in a proteinaceous shell [6]. Viruses display an enormous variability in terms of genomes (comprising nucleic acids made of either DNA or RNA, either single or double‐stranded, with linear or circular molecules present as a single element or segmented into different entities) and morphologies (with or without an envelope, with either icosahedral or helical symmetry) [7]. Viruses are highly relevant not only for clinical reasons but also for their impact on agriculture and biodiversity [8].

**FIGURE 2:**
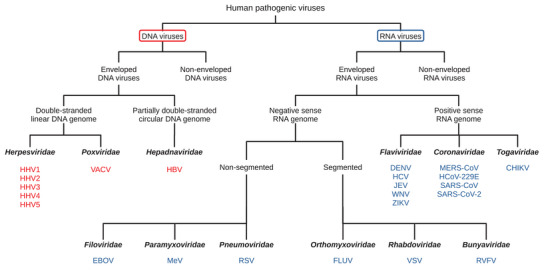
Overview of the viruses reported in the present review. Viruses are stratified by genome (DNA or RNA), presence or absence of an envelope, and type of genome (single‐ or double‐stranded, positive or negative sense). The families of the viruses reported in this review are indicated together with the species. CHIKV, chikungunya virus; DENV, dengue virus; EBOV, Ebola virus; FLUV, influenza virus; HBV, hepatitis B virus; HCoV, human coronavirus; HCV, hepatitis C virus; HHV, human herpesvirus; JEV, Japanese encephalitis virus; MERS‐CoV, Middle East respiratory syndrome coronavirus; MeV, measles virus; RSV, respiratory syncytial virus; RVFV, Rift Valley fever virus; SARS‐CoV, severe acute respiratory syndrome coronavirus; VACV, vaccinia virus; VSV, vesicular stomatitis Indiana virus; WNV, West Nile virus; ZIKV, Zika virus.

It would be impossible to summarize the whole body of knowledge on the antiviral action of polyphenols, and there are several excellent reviews on the subject [7]. The present review will instead provide an overview of the most characterized molecular mechanisms involved in the hindrance of viral infection associated with the use of polyphenols, focusing on human pathogens belonging to enveloped viruses (Figure 2 and Tables 2, 3).

**TABLE 2:** Summary of antiviral activity of polyphenols reported in the present review.

Compound	Group	Source	Dosage^a^	Virus	Genome^b^	Pathway affected	Ref.
Baicalin	Flavonoids	*Scutellaria baicalensis*	−	HBV	dsDNA	Replication	[69, 70]
		*Radix scudellariae*	−	HBV	dsDNA	Transcription	[71]
Caffeic acid	Phenolic acids	*Solanum melangena*	27.2 µM	HHV1	dsDNA	Transcription	[31]
Chebulagic acid	Tannins	*Terminalia chebula*	1.41 µg/mL	HHV2	dsDNA	Attachment	[26]
Chebulinic acid	Tannins	*Terminalia chebula*	0.06 µg/mL	HHV2	dsDNA	Attachment	[26]
Chlorogenic acid	−	−	−	EBOV	(–)RNA	Attachment	[90]
Curcumin	Curcuminoids	−	1.90 µM	ZIKV	(+)RNA	Virion structure	[176]
		−	3.89 µM	CHIKV	(+)RNA	Virion structure	[176]
		−	−	HCV	(+)RNA	Virion strucutre	[143]
EGCG	Flavonoids	*Camellia sinensis*	10 µM	HBV	dsDNA	Viral entry	[74]
		−	21.4 µM	ZIKV	(+)RNA	Viral entry	[129]
		Synthesized	0.73 µM	ZIKV	(+)RNA	Transcription	[131]
		*Limonium densiflorum*	19 µg/mL	FLUV	(–)RNA	Virion structure	[106]
		−	−	EBOV	(–)RNA	Attachment	[90]
Oleuropein	−	−	−	EBOV	(–)RNA	Attachment	[90]
Gallic acid	Phenolic acids	*Graptopetalum paraguayense*	0.92 µg/mL	HHV1 HHV2	dsDNA	Replication	[29]
Geraniin	Tannins	*Spondias mombin*		HHV1	dsDNA	Attachment	[27]
Ginkgolic acid	Phenolic acids	*Ginkgo biloba*		HHV1	dsDNA	Translation	[24]
		−	−	HHV5	dsDNA	Viral entry	[24]
		−	−	HHV1	dsDNA	Viral entry	[25]
Glycyrrhetinic acid	−	*Glycyrrhiza glabra*	0.13–0.56 µM	ZIKV	(+)RNA	NS1 inhibition	[132]
Honokiol	Lignans	*Magnolia* spp.	10.51 µg/mL	HHV1	dsDNA	Replication Transcription	[39]
Hyperoside	Flavonoids	*Abelmoschus manihot*	−	HBV	dsDNA	−	[65]
Imperatorin	Coumarin	*Angelica archangelica*	−	HHV1	dsDNA	Replication	[37]
Luteolin	Flavonoids	*Swertia macrosperma*	20 µM	HBV	dsDNA	−	[68]
		Synthesized	0.07 µM	FLUV	(–)RNA	Replication	[100]
Manassantin	Lignans	*Saurus chinensis*	12.96 µM	HHV4	dsDNA	Transcription	[177]
Miquelianin	−	−	−	EBOV	(–)RNA	Attachment	[90]
Pentagalloylglucose	Tannins	*Elaeocarpus sylvestris*	14.67 µg/mL	HHV3	dsDNA	Modulation c‐Jun Transcription IE62	[42]
Phellopterin	Coumarin	*Angelica archangelica*	−	HHV1	dsDNA	Replication	[37]
Piceatannol	Stilbenes		−	HHV5	dsDNA	Transcription P16*^INK4^* ^a^ downregulation	[43]
Pinocembrin	Flavonoids	−	17.4 µM	ZIKV	(+)RNA	Replication Translation	[138]
		−	21.5 µM	CHIKV	(+)RNA		[138]
		−	39.0 µM	DENV	(+)RNA		[138]
Protocatechuic acid	Phenolic acids	*Hibiscus sabdariffa*	−	HHV1 HHV2	dsDNA	Replication	[30]
Psiloxylon	−	*Psiloxylon maurititianum*	100 µg/mL	DENV ZIKV	(+)RNA	Attachment	[149]
Punicalagin	Tannins	*Punica granatum*	−	HHV2	dsDNA	Viral protease	[32]
Pyrogallol	−	Synthesized	30 µg/mL	FLUV	(–)RNA	Oxidative stress	[104]
Quercetin	Flavonoids	Synthesized	1.17 µM	ZIKV	(+)RNA	Transcription	[131]
		Synthesized	0.67 µM	FLUV	(–)RNA	Replication	[100]
		*Rapanea melanophloeos*	150 µM	FLUV	(–)RNA	IFN modulation	[102]
Quinin	−	Synthesized	150 µM	DENV	(+)RNA	Replication	[148]
Resveratrol	Stilbenes	Berries, legumes	−	HHV1	dsDNA	IE inhibition	[34]
			−	HHV2	dsDNA	Oxidative stress	[35]
		*Vitis vinifera*	0.9 µg/mL	HHV1	dsDNA	Replication	[38]
			−	HHV4	dsDNA	Transcription	[40]
			40 µg/mL	FLUV	(−)RNA	Transcription Nuclear exportation	[105]
		Synthesized	3.5–4.7 µM	VACV	dsDNA	Replication	[178]
Rosmarinic acid	Phenolic acids	−	30 µM	HBV	dsDNA	Replication	[78]
Rutamarin	Coumarins	*Ruta graveolens*	13.8 µM	HHV4	dsDNA	Replication Translation	[28]
Silymarin	−	Synthesized	100 µg/mL	CHIKV	(+)RNA	Replication	[134]
Syringic acid	Phenolic acids	*Graptopetalum paraguayense*	0.92 µg/mL	HHV1 HHV2	dsDNA	Replication	[29]
TCQA	−	*Ilex pubescens*	200 µM	FLUV	(–)RNA	IFN modulation	[103]
TGG	−	−	−	EBOV	(–)RNA	Attachment	[90]
*Trans*‐ferulic acid	Phenolic acids	*Graptopetalum paraguayense*	0.92 µg/mL	HHV1 HHV2	dsDNA	Replication	[29]
Vanillic acid	Phenolic acids	*Graptopetalum paraguayense*	0.92 µg/mL	HHV1 HHV2	dsDNA	Replication	[29]
Wogonin	Flavonoids	*Scutellaria baicalnsis/radix*	−	HBV	dsDNA	−	[66, 67]
Xanthohumol	Chalcon	*Humulus lupulus*	162 µM	SARS‐CoV‐2	(+)RNA	Inhibition PL^pro^	[156]

^a^ Concentration used in the study, including IC_50_ or EC_50_.

^b^ Legend: ds = double‐stranded; p = partial double‐stranded; (+) = positive‐strand; (–) = negative‐strand.

**TABLE 3:** Overview of the antiviral activity of polyphenols class.

Virus	Phenolic acid	Stilbenes	Lignans	Coumarins	Flavonoids	Tannins
Arboviruses	−	−	−	−	ZIKV	−
Coronaviruses	−	−	−	−	SARS‐CoV‐2	−
Filoviridae	EBOV	−	−	−	−	−
Hepadnaviruses	−	−	−	−	HBV	−
Herpesviruses	HHV1, HHV2	HHV1, HHV2	HHV1	HHV1	−	HHV1, HHV2
Poxviruses	−	VACV	−	−	−	−

## 2 DNA Viruses

### 2.1 Double‐Stranded Linear DNA Viruses

#### 2.1.1 Overview

The group of double‐stranded linear DNA viruses includes the *Herpesviridae* family, which is formed by three subfamilies, each containing clinically relevant genera (Figure 2): *Alphaherpesvirinae* with human herpes simplex virus (human herpesvirus [HHV]1 and HHV2) and varicellovirus (HHV3, previously known as varicella‐zoster virus); *Betaherpesvirinae* with HHV5 (previously cytomegalovirus) and roseolovirus (HHV6 and HHV7); and *Gammaherpesvirinae* with lymphocryptovirus such as HHV4 (previously Epstein–Barr virus) [8]. The herpesviruses can cause localized mucocutaneous lesions, meningitis, and encephalitis (HHV1, HHV2) and are associated with various malignancies, including lymphomas like Hodgkin's disease (HHV4) [9]. Another relevant viral clade within the group is that of the poxviruses (*Poxviridae* family), with smallpox, monkeypox, and vaccinia virus (VACV) as the most clinically significant species [10].

The *Herpesviridae* share the feature of a large (over 150 kb in length) double‐stranded DNA genome encapsulated into an icosahedral capsid further embedded into a proteinaceous tegument (called matrix in other viruses) and a phospholipid envelope [9]. The standard herpesvirus treatment is aciclovir, which interferes with the viral DNA polymerase [11]. Since resistance to this drug is becoming increasingly frequent, polyphenols have gained the spotlight as possible antiherpetic alternatives [12].

**FIGURE 3:**
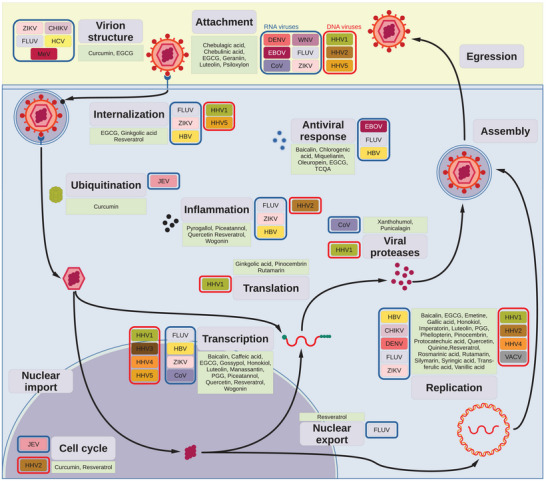
Summary of the antiviral activity of polyphenols. Overview of the viral infection pathways affected by polyphenols. The main aspects of the viral infection are depicted (round boxes) together with the polyphenols involved in the hindrance (green boxes) and the viruses affected (colored boxes). Viruses are further stratified by their genome (DNA or RNA) by a colored box (red or blue, respectively). CHIKV, chikungunya virus; CoV, coronavirus; DENV, dengue virus; EBOV, Ebola virus; FLUV, influenza virus; HBV, hepatitis B virus; HCV, hepatitis C virus; HHV, human herpesvirus; JEV, Japanese encephalitis virus; MeV, measles virus; VACV, vaccinia virus; WNV, West Nile virus; ZIKV, Zika virus.

Attachment and entry of herpesviruses are best elucidated in HHV1; these steps are mediated by a complex of the surface glycoproteins gB, gD, gH, and gL (Figure 3) [13]. The entry process begins with binding the herpesvirus entry mediator (HVEM), a ubiquitous tumor necrosis factor receptor superfamily member [14]. The viral particle is internalized within endocytic vesicles and released into the cytoplasm [15]. The viral genomes are then released inside the nucleus [16], where the viral genes are transcribed by the cellular RNA polymerase II (RpII) [17]. Viral replication does not occur immediately; the virus establishes a latency phase. The tegument viral protein VP16 is an essential factor in this process: it forms a complex with the cellular transcription factor Oct‐1 and the transcription Factor II D (TFIID) that directs RpII to the viral immediate‐early (IE) promoters [18]. VP16 additionally promotes latency by facilitating heterochromatin formation within the viral chromosome [19]. VP16 also hampers the antiviral cellular response by downregulating the expression of type one interferon (IFN‐I) [20]. Infected‐cell protein 22 (ICP22) also hinders IFN‐I [21]. In addition, viral protein 16 (VP16) facilitates the assembly of new capsids, which are then released by budding into the perinuclear space as enveloped virions [22].

Herpesviruses also counteract the apoptosis activated by viral infection, a feature particularly relevant in HHV4. For instance, the latent membrane protein 1 (LMP‐1) is a *trans*‐activator of several cellular pathways, such as the nuclear factor kappa B (NF‐κB) that inhibits the apoptotic signal [23].

#### 2.1.2 Polyphenol Applications

It has been reported that ginkgolic acid from *Ginkgo biloba* impaired HHV1, HHV4, and HHV5 entry by hindrance of gB activity [24, 25], whereas geraniin from *Spondias mombin* and chebulagic/chebulinic acid from *Terminalia chebula* inhibited HHV1 and HHV2 attachment to host cells, respectively [26, 27]. Ginkgolic acid also inhibited HHV1 proteins involved in the translation step, thus hampering virion production [24]. Protein synthesis in HHV4 was inhibited by rutamarin from *Ruta graveolens* [28]. In contrast, a plethora of polyphenol molecules such as protocatechuic acid from *Hibiscus sabdariffa* as well as *trans*‐ferulic acid, vanillic acid, syringic acid, and gallic acid from *Graptopetalum paraguayense* could interfere with the replication of HHV1 and HHV2 genomes, mainly through the hindrance of DNA polymerase activity [29, 30].

Polyphenols from *Solanum melangena* were shown to inhibit viral DNA replication by downgrading the expression of HHV1 gB [31]. Punicalagin from *Punica granatum* inhibited HHV2 protease [32]. HHV1 was also hampered by resveratrol, one of the main active molecules in grapes (*Vitis vinifera*), which has antiinflammatory, immune‐modulatory, and antiaging capabilities [33]. This polyphenol has been suggested to inhibit the transcription of HHV1 viral IE genes [34] and, in HHV2, modulate the expression of NF‐κB and cyclin‐dependent kinase 9 (CDK‐9) [35]. A preparation from *Geranium sanguineum* inhibited HHV1 and HHV2 with a 50% inhibitory concentration (IC_50_) of 3.6–6.2 µg/mL [36].

Resveratrol extracted from grape cane (*V. vinifera*), as well as imperatorin and phellopterin from *Angelica archangelica*, could also inhibit HHV1 replication [37, 38]. Downregulation of HHV1 ICP27 and VP16 gene expression was demonstrated using honokiol from *Magnolia* spp., boosting aciclovir activity [39]. Resveratrol could also downregulate the expression of HHV4 replication and transcription activation (Rta) [40]. Further, resveratrol inhibited viral replication and load in piglets as measured by nasal swabs and in brain tissue. Moreover, serum IFN‐III was also elevated [41].

Pentagalloylglucose (PGG) from *Elaeocarpus sylvestris* inhibited HHV3 replication by downgrading the viral‐driven activation of c‐Jun signaling pathway and the expression of the viral modulator IE62 [42]. Piceatannol could block the expression of HHV5 IE genes and downregulate the viral‐driven activation of p16*^INK4a^* [43]. Massantin from *Saurus chinensis* can inhibit HHV4 BamHI Z fragment leftward open reading Frame 1 (BZLF1) expression [56].

### 2.2 Partially Double‐Stranded Circular DNA Viruses

#### 2.2.1 Overview

The group of partially double‐stranded circular DNA viruses contains comparatively few representatives (Figure 2). However, the human hepatitis B virus (HBV, *Hepadnaviridae*) represents a significant source of morbidity and mortality despite the availability of an effective vaccine likely to progress toward chronic B hepatitis (CBH), which causes over 800 000 deaths yearly [44]. CBH treatment is mainly based on administering nucleotide and nucleoside analogs, such as lamivudine and adefovir, which reduce the viral load in infected cells [45]. However, a substantial proportion of treated patients, ranging 13%–39%, developed resistance to the drugs [46]. Hence, plant‐based alternatives are actively sought as novel treatments for CBH.

The human HBV genome comprises a linear chain of 3.2 kb in length of negative‐sense polarity, or (−)DNA, circularized for the presence of terminal inverted repeats [47]. The 5′‐end of this strand is also covalently linked to a tyrosine residue of the viral polymerase P [48], a positive‐sense fragment of variable length [49], and an RNA segment leftover of the degradation of a full‐length RNA known as pre‐genomic (pgRNA) [50]. The pgRNA is enclosed within an icosahedral shell 22 nm in diameter composed of a capsid protein with an antigenic activity called HBV core antigen (HBcAg) [51]. This particle is further surrounded by an envelope decorated with viral protein that also elicits a high immune response and is termed HBV surface antigens (HBsAgs) [52].

HBV gains access to hepatocytes by binding the Na*^+^*/taurocholate co‐transporter polypeptide (NTCP), which mediates the import of bile acids inside the hepatocytes (Figure 3) [53]. The viral genome is then released into the nucleus [54], where the cellular DNA repair machinery detaches P, seals the nicks, and produces the complementary strand by extending the small regions of dsDNA to produce a fully functional viral episome [55]. The hepatitis B viral protein X (HBx) ensures proper transcription from the viral genome by epigenetic means [56].

Among the most relevant proteins encoded by HBV is the hepatitis B extracellular antigen (HBeAg), which is secreted and inhibits the activation of the NF‐κB and IFN‐γ pathways [57]. The weakening of the immune response is associated with the inability of the host to clear the infection and a higher risk of developing chronic infections with associated hepatitis [58]. Another essential viral protein is the HBsAg, the primary marker for HBV infection [59].

The pgRNA chain is bound by P through the recognition of a sequence (ε) of about 70 nucleotides and the complex is encapsulated by the core protein HBcAg [60]. Viral transcription requires a series of cellular factors, including the farnesoid‐X‐receptor (FXR), which modulates the expression of several genes involved in bile metabolism [61]. P binding to ε activates the enzyme's reverse‐transcription activity [62]. Subsequently, P's ribonuclease H (RNaseH) activity is engaged, leading to pgRNA degradation, except for the last 18 nucleotides [63]. The leftover pgRNA is used as a primer for synthesizing the (−)DNA strand and then used as a template to produce (+)DNA strands of different lengths, known as replicative intermediates [64].

#### 2.2.2 Polyphenol Applications

It was widely reported that polyphenols can have anti‐HBV activity. For instance, hyperoside from *Abelmoschus manihot* significantly decreased serum HBV DNA as well as the level of liver damage in Peking ducklings compared to controls [65]. Moreover, wogonin from *Scutellaria baicalensis* significantly suppressed HBsAg expression in a dose‐dependent manner in the hepatocellular cell line MS‐G2 [66]. Wogonin from *Scutellaria radix* also decreased plasma HBV DNA load, and hepatic necrosis and inflammatory cell infiltration in ducks and reduced serum HBsAg in human HBV‐transgenic mice compared to controls [67]. Luteolin from *Swertia macrosperma* could decrease the expression of HBsAg and HBeAg in exposed HepG2.2.15 cells [68].

HBsAg, HBeAg, and viral loads were also reduced by baicalin from *S. baicalensis* in hepatic cells constitutively expressing HBV (HepG2, HepG2.2.15, and Huh7) [69, 70]. The authors suggested that baicalin could suppress viral RNA replication. In another report, baicalin from S. radix *Radix scudellariae* stimulated the expression of IFN‐I/III in microvascular endothelial cells [71]. Since IFN is known to inhibit the activation of the NF‐κB signal pathway essential for HBV activation [72], baicalin might hinder viral gene expression leading to a reduction in viral markers of infection.

The mechanism of action of polyphenols against HBV has been characterized to a greater extent for epigallocatechin‐3‐gallate (EGCG), one of the main components of green tea (*Camellia sinensis*) [73]. Exposure of immortalized primary human hepatocytes containing HBV (HuS‐E/2 cell line) to EGCG (Sigma–Aldrich) resulted in a significant reduction in intracellular viral RNA load and viral infectivity as well as an eight‐fold decrease in the proportion of cells expressing HBV core proteins [74]. The authors of this study also showed a reduction in the internalization of NTCP and transferrin, which is also internalized by a clathrin‐mediated process [75], upon EGCG exposure while, at the same time, HBV replication and virion production were not affected [74]. Conversely, studies performed on HBV‐containing cell lines HepG2.117 showed that exposure to EGCG decreased the synthesis of replicative intermediates of the DNA genome [76]. Others have shown that EGCG binds FXR in HBV‐expressing cell line HepG2‐N10, hampering the activation of viral genes [77].

Rosmarinic acid hampers the binding of P to the ε sequence, causing a decrease in viral load and HBsAg expression in HEK‐293T and HepG2 cells transfected with HBV‐containing plasmids [78].

## 3 Negative Sense Single‐Stranded RNA Viruses

### 3.1 Nonsegmented Genomes

#### 3.1.1 Overview

The main characteristic of negative‐sense single‐stranded viruses is that the genome comprises a single molecule used as a template for producing the mRNAs encoding the viral proteins [79, 80]. One of the prominent members of this group is the measles virus (MeV) (Figure 2), which belongs to the genus *Morbillivirus* (family *Paramyxoviridae*; subfamily *Orthoparamyxovirinae*) and has a diameter of about 100–300 nm whose prevalence has been increasing in recent years [81]. MeV is highly transmissible and can cause many symptoms, from skin rash to encephalitis and immune suppression [82].

The virus enters the organism through the respiratory tract but initially does not infect epithelial cells (Figure 3). Instead, it is taken up by dendritic cells and macrophages expressing the signaling lymphocytic activation molecule (SLAM) receptor on their surface [81]. Lymphocytes are also infected after binding the MeV hemagglutinin (HA) protein to the target entry receptor, triggering the modification of the fusion protein (F). MeV uses lymphocytes to infect different tissues, and the new virions infect the respiratory tree epithelial cells by using the nectin‐4 receptor, resulting in their destruction that, in turn, causes intense cough with virus‐containing droplets [81].

In the infected cells, the cellular RNA‐dependent RNA‐polymerase (RdRp) initiates the transcription from the viral genome, generating mRNAs and encoding for the viral proteins [81]. During the replication process, the C protein is recruited to the ribonucleocapsid complex supposedly increasing polymerase activity.

Ebola virus (EBOV) is another clinically relevant virus belonging to the group that, unlike MeV, is only present endemic in certain parts of Africa [83]. The largest outbreak of EBOV disease was observed in 2014 in West Africa since the first outbreak in 1976 [84]. EBOV is a highly pathogenic agent associated with acute hemorrhagic fever with a mortality rate of 60%–90% [85]. EBOV belongs to the genus Orthoebolavirus (family *Filoviridae*) and has a helicoidal symmetry with a diameter of about 80 nm and a 970–1200 nm length. The helical nucleocapsid is surrounded by an outer envelope with anchored specific glycoprotein spikes involved in attachment and entry [84]. The Zaire Ebola viral matrix protein VP24, located between the nucleocapsid and the outer envelope [84], is involved in preventing the interferon‐induced antiviral responses by inhibiting the nuclear import of signal transducer and activator of transcription 1 (STAT1).

EBOV can replicate in many human cells, including immune cells, endothelial cells, fibroblasts, hepatocytes, and adrenal cells. EBOV enters the organism through the viral envelope glycoprotein, which is responsible for receptor binding and fusion of the viral envelope with the host membrane [86]. The EBOV targets macrophages, dendritic cells, and endothelial cells and also affects the gastrointestinal (GI) tract (diarrhea), the adrenal glands, and the liver (multifocal necrosis, decreased production of clotting factors) [87]. The virus infection first appears to disable the immune system and subsequently disables the vascular system, which leads to blood leakage (hemorrhage), hypotension, and a drop in blood pressure, followed by shock and death [87].

#### 3.1.2 Polyphenol Applications

Polyphenols (5 µg/mL) extracted from the seaweed *Ecklonia arborea* and *Solieria filiformis* reduced MeV infectious titers in Vero cells, with negligible cytotoxicity, through a mechanism that involved the alteration of the capsid [88].

Polyphenols extracted from *Cistus incanus* (Ci) display broad‐spectrum antiviral activity against multiple unrelated, major human viral pathogens like EBOV with low risk of virus resistance. The mechanism blocks the virus attachment to host cells by selectively targeting the viral envelope glycoproteins. In contrast, other natural products with broad‐spectrum antiviral activity target host cell components [89].

Polyphenols like 1,2,3,6‐tetragalloyl glucose (TGG), epigallocatechin gallate, chlorogenic acid, oleuropein, and miquelianin may neutralize the threatening action of EBOV by inhibiting of VP24 [90]. Emetine inhibited the infection of HeLa and Vero cells with virus‐like particles of EBOV with IC_50_ of 10.2 and 0.017 µM, respectively [91].

### 3.2 Segmented Genomes

#### 3.2.1 Overview

Segmented genomes characterize this group of viruses (Figure 2), a feature indicating that the virion includes several genomic molecules (segments); infectious particles must contain at least one copy of each segment [80]. Although such a strategy might decrease infectivity, it increases the genetic fluidity of the virus by allowing for the phenomenon of recombination and expanding the actual size of the genome [92].

Influenza virus (FLUV) belongs to the *Orthomyxoviridae* family and is a significant cause of morbidity and mortality worldwide, despite the availability of vaccines and antivirals [93]. Genotype A (FLUV‐A, *Alphainfluenzavirus* genus), is the most clinically relevant of the family [94]. FLUV virions consist of enveloped particles of about 100 nm in diameter containing a matrix encapsidating eight nucleoprotein segments [95].

The viral entry is mediated by the HA protein, which binds to the sialic acid *N*‐acetylneuraminic acid (Neu5Ac), a common element of human cell membranes (Figure 3) [96]. Endocytosis is clathrin‐mediated [91] and HA is also responsible for releasing the viral particles into the cytoplasm [92]. After dissociation of the matrix, the nucleocapsids and the viral RdRp are imported into the nucleus where the viral polymerase will transcribe the influenza genes [97]. Newly synthesized viral components accumulate at the plasmalemma, where the virions egress by budding [98]. Since HA in the released virion will interact with Neu5Ac, causing reinfection of the already infected cell, FLUV has developed a specific protein, the neuraminidase (NA), that removes the sialic acid from HA, freeing the newly synthesized virions [99].

#### 3.2.2 Polyphenol Applications

Quercetin and luteolin exhibited stark inhibitory activity against FLUV‐A RdRp with an IC_50_ of 0.67 and 0.07 µM, respectively [100]. Quercetin and chlorogenic acid from *Honeysuckle* spp. increased the survival of BALB/c mice by 16% and 20%, respectively [101]. A quercetin derivative (quercetin‐3‐*O*‐α‐l‐rhamnopyranoside) from *Rapanea melanophloeos* supplemented at a concentration of 150 µM reduced the expression of the proinflammatory cytokine IL‐6 in canine kidney cells MDCK exposed to FLUV‐A, suggesting a reduction in the pathogenic activity of the virus [102].

An extract from *Ilex pubescens*, 3,4,5‐tri‐*O*‐caffeoylquinic acid (TCQA), was shown to be active against FLUV‐A in human lung epithelial cells H292: cells exposed to 200 µM showed lower infectious foci and altered genetic expression compared to unexposed controls [103]. In particular, TCQA increased the transcription of genes involved in the antiviral response, such as type I IFN, but decreased the activation of signal pathways abnormally stimulated during FLUV‐A infection, such as toll‐like receptor 3 (TLR3) and interferon regulatory Factor 7 (IRF7). Pyrogallol at a concentration of 30 µg/mL showed an IC_50_ ranging from 0.70 to 1.48 µg/mL according to the FLUV‐A strain in an MDCK cells model [104]. The authors of this study reported that the treatment caused an increase in heme oxygenase‐1 (HO‐1) expression that, in turn, induced a reduction in the oxidative stress to the cell and the cytopathic effect linked to viral infection.

Resveratrol supplemented at a concentration of 10–40 µg/mL reduced FLUV‐A titers compared to controls, while cytopathic effects were absent at concentrations below 20 µg/mL [105]. Pretreatment of cells with this molecule did not alter viral infectivity indicating that resveratrol acted in steps following the entry phase. In particular, resveratrol did not inhibit viral RNA synthesis but their transcription and export of viral proteins from the nucleus [105].

EGCG from *Limonium densiflorum* showed an IC_50_ of 19 µg/mL against FLUV‐A in MDCK cells with a mechanism supposed to involve the attachment and alteration of the virion [106]. Other studies reported that the mechanism of action of polyphenols is linked to the attachment phase. Polyphenols from *Saccharum officinarum* inhibited FLUV‐A replication in MCDK cells in a dose‐dependent fashion, with a 95% reduction in viral titers obtained at a concentration of 1 mg/mL while displaying negligible cytotoxicity [107]. The authors of this study showed that the inhibition was associated with the hindrance of HA binding to sialic acid. A preparation from *G. sanguineum* inhibited H1N1 and H7N1 with a 50% effective concentration (EC_50_) of 2.2 and 2.1 µg/mL, respectively [36].

## 4 Positive Sense Single‐Stranded RNA Viruses

### 4.1 Overview

The group of single‐stranded positive‐sense RNA viruses is characterized by genomes that can be used directly as transcripts to produce viral proteins [108]. The group includes several human pathogens including the *Coronaviridae* family, a clade the comprise the etiological agent of the COVID‐19 pandemic (the severe acute respiratory syndrome coronavirus [SARS‐CoV]‐2), other viruses characterized by high mortality and transmissibility (SARS‐CoV and the Middle East respiratory syndrome coronavirus [MERS‐CoV]) as well as nonsevere acute respiratory syndrome viruses human coronavirus (HCoV) 229E (Figure 2) [109]. Another major class within the group encompasses the viruses transmitted by arthropods (arboviruses) [110], with the family *Flaviridae* having a special clinical importance because it incorporates severe human pathogens with wide distribution, such as Zika virus (ZIKV), dengue virus (DENV), West Nile virus (WNV), and Japanese encephalitis virus (JEV) [111]. Another relevant arbovirus is the chikungunya virus (CHIKV), which belongs to the *Togaviridae* family [112]. In addition, the *Flaviviridae* family includes hepatitis C virus (HCV), which is not transmitted by arthropods [113].

The group of single‐stranded positive‐sense RNA viruses has a high potential for generating pandemics. ZIKV is currently endemic in 86 countries [114] and, in 2016, the World Health Organization declared ZIKV a global public health concern [115]. By comparison, DENV has been reported in 141 countries around the world [116]. COVID‐19 is a still ongoing pandemic that has so far killed more than 7 million people worldwide since its outbreak in 2019 [117]. Moreover, treatments for these viruses are still missing. There are neither vaccines nor drugs to treat or prevent ZIKV infections [118].

The flaviviruses share the feature of an enveloped capsid formed by the capsid (C), envelope (E), and membrane (M) proteins surrounding a genome composed of positive‐sense single‐stranded RNA that is 3´‐capped with conserved secondary structures at both ends of the genome [119]. The attachment to the host cell is initiated by binding the E protein to surface glycosaminoglycans (Figure 3) [120]. Once released into the cytoplasm, the genome is recognized as a bona fide mRNA and is translated by cellular transcription machinery. The viral genome contains a single ORF encoding a single polyprotein cleaved by several cellular peptidases and the viral serine protease nonstructural protein 3 (NS3) [121]. NS1, NS3, and NS5 are of particular interest. NS1's primary function is that of a scaffold protein to form the replication complex [122]. NS1 interacts with many cellular factors and is the only excreted viral protein in the form of a soluble homo‐hexamer (sNS1) [123]. Secreted NS1 modulates the immune response and triggers autoimmune reactions and vascular permeability [124]. NS3 is the viral serine protease [125] and NS5 is the RdRp [126]. The coronaviruses also produce a single polyprotein that is matured by cleavage [127].

### 4.2 Polyphenol Applications

Polyphenols have been shown to affect essentially all aspects of the ZIKV infection cycle. For instance, EGCG supplied in a 1–10 µM concentration range inhibited viral adsorption and internalization of ZIKV, DENV, and WNV [128]. Regarding the inhibition of ZIKV entry, EGCG showed a strain‐specific activity: isolate ZIKVBR was inhibited by 50 µM of EGCG, but ZIKV MR766 required only 25 µM; the EC_50_ was calculated as 21.4 µM [129]. Cell toxicity was reported for exposures of at least 200 µM [129]. EGCG (from Sigma–Aldrich) hampered ZIKV nucleoside‐triphosphatase (NTPase) with an IC_50_ 0.296 µM in an enzymatic assay [130].

Both quercetin and EGCG inhibited NS3 activity with an EC_50_ of 1.17 and 0.73 µM, respectively [131], while glycyrrhetinic acid, one of the main active molecules of licorice (*Glycyrrhiza glabra*) inhibited NS1 [132], and extracts from *Andrographis paniculata* impaired RNA replication by hampering NS5 [133]. Silymarin, the main component of *Silybum marianum*, was shown to be even more effective than quercetin in inhibiting CHIKV replication, with a concentration of 100 µg/mL, whereas quercetin showed no significant reduction of viral titers [134].

Resveratrol (from Sigma–Aldrich) inhibited ZIKV replication in Vero and Huh7 cells [135]. Emodin was effective against ZIKV but also many other viruses [136]. Further, emetine was characterized as an inhibitor of ZIKV replication (IC_50_: 8.74 nM) that also decreases viral entry. NS1 levels declined in the presence of emetine (IC_50_: 52.9 nM) [91].

Pinocembrin, which is one of the main active components of propolis [137], was used against ZIKV in hamster (BHK‐21), human embryonic kidney (HEK293T), human carcinoma (Huh7), and human epithelial (JEG‐3) cell lines [138]. This study determined the 50% cytotoxic concentration (CC_50_) and IC_50_ in 251 and 17.4 µM. Interestingly, pinocembrin treatment (19.5 or 39.0 µM) was effective against ZIKV only in Huh7 cells without affecting cell viability. In addition, pretreatment of JEG‐3 cell with pinocembrin at a concentration of 156 µM 2 h before infection did not affect viral replication, while postinfection treatment inhibited the synthesis of viral RNA as well as envelope proteins, indicating that this molecule interfered with the virus after the attachment step [138]. Moreover, pinocembrin was also active against other flaviviruses. For instance, treatment of Huh7 cells with 39 µM and HeLa cells with 21.5 µM of pinocembrin determined a significant reduction of DENV and CHIKV titers compared to untreated controls [138].

Curcumin is well tolerated by human cells, but its low bioavailability limits its use [139]. It has been employed against DENV2 (EC_50_; 13.95 µM, CC_50_: 49.01 µM, selectivity index [SI]: 3.51) determined by plaque formation assay, but also against respiratory syncytial virus (RSV), vesicular stomatitis Indiana virus (VSV), ZIKV, FLUV, and HCV [140, 141, 142, 141, 143]. Curcumin could also weakly inhibit the viral protease (IC_50_: 66.01 µM), the expression of genes related to lipid biosynthesis, and it further inhibited actin polymerization [140]. Several curcuminoids have been tested against DENV2, and many of them showed even higher activity in plaque‐forming assays than their parent compound, curcumin [140].

Curcumin (from Sigma–Aldrich) showed a dose‐dependent reduction in ZIKV titers, with 5 µM being the minimal inhibiting concentration, which was below the amount needed to markedly decrease cell viability (10 µM) [142]. Unlike pinocembrin, curcumin treatment (5 µM) was effective against ZIKV and CHIKV both before and after infection, although pretreatment showed the strongest inhibitory effect [142]. Interestingly, curcumin did not affect the accumulation of viral RNA or proteins after infection, indicating that the molecule affected an early stage of the infection process.

Since curcumin induced alteration in vesicle fluidity, reducing the attachment rate and entry into human liver cells of all tested genotypes of HCV [143], the authors suggested a similar mechanism to explain the observed ZIKV repression [142]. Interestingly, curcumin did not downregulate HCV replication and reduced the infectivity of HCV without disrupting the integrity of the virus particles [143].

Nonetheless, this mechanism might not be the only one carried out by this curcumin. It has been suggested that the reduction in JEV infectious particles observed while treating porcine kidney cells with 5 µM curcumin could be due to the increased ubiquitination that possibly removed, along with other proteins, viral products from the cytoplasm [144] and decreasing NF‐κB activation in Rift Valley fever virus (RVFV) infections [145]. Curcumin, EGCG, and resveratrol inhibit the NF‐κB pathways [146].

Quinine has been used for centuries as an anti‐malaria treatment, although in recent times, the frequency of resistant infection has increased, and plant‐based alternatives are actively sought [147]. Its administration at a concentration of 150 µM reduced DENV viral load in three human cell lines (HepG2, A549, and EA.hy926) in a dose‐dependent manner, with an increase in the expression of showing an increase in IFN‐I, 2′‐5′‐oligoadenylate synthetase 3 (OAS‐3), RNase L, and protein kinase RNA‐activated (PKR) compared to untreated controls, suggesting that this molecule interfered with viral replication in several points [148].

Psiloxylon from *Psiloxylon maurititianum* showed a CC_50_ of 1044 µg/mL and caused a reduction in both DENV and ZIKV's viral replication at a 100 µg/mL concentration [149]. In particular, exposure to psiloxylon concomitantly to infection resulted in a significant reduction of infected cells but pretreatment was ineffective, suggesting that this molecule acted in the attachment phase of infection. The authors of the study demonstrated that exposure to extracts of *P. mauritianum* caused a ten‐fold reduction in the number of particles attached to host cells [149].

Polyphenols also showed the capability of disrupting other targets than the viral proteases. For instance, in silico analysis identified several plant derivatives capable of binding SARS‐CoV‐2 RdRp, particularly theaflavin, hesperidin, EGCG, myricetin, and quercetagetin [150]. There are several plant‐derived molecules active against SARS‐COV‐2; we refer to more exhaustive reviews for further information [151], and we focus on some of the most effective reported so far.

Resveratrol is active against FLUV‐A, RSV, SARS‐CoV, and MERS‐CoV [152]. Neutrophils isolated from COVID‐19 patients and exposed to 100 µM resveratrol (*Sigma–Aldrich*) showed reduced liberation of DNA, suggesting a reduced activation of these cells [33]. In SARS‐CoV‐2, resveratrol was found to hamper viral replication, the viral main protease (M^pro^) (IC_50_: 16.5 µM) [153, 154].

In our laboratory, we experimentally established that xanthohumol (XN), a prenylated chalcone produced by the female hop cones [155], impaired the SARS‐CoV‐2 papain‐like protease (PL^pro^). Our docking analysis suggested that XN could block the PL^pro^ binding site to the viral polyprotein; such hindrance resulted in a IC_50_ values of 3.3 µM [156]. In particular, our docking analysis showed that all these molecules could bind to the active site of the viral PL^pro^, an enzyme involved in the maturation of the viral polyprotein precursors and, therefore, a suitable target for antiviral therapy [157]. The theoretical hindrance determined by molecular docking was confirmed by our enzymatic testing, which showed a reduction of PL^pro^ activity in cleaving the reporter protein interferon‐stimulated gene 15 (ISG15).

Resveratrol also inhibited SARS‐CoV_2 viroporin ORF3a (IC_50_: 6.7 µM) [153, 154]. Channel activity of ORF3a was also inhibited by kaempferol (IC_50_: 5.2 µM), quercetin (IC_50_: 2.5 µM), EGCG (IC_50_: 1.6 µM), nobiletin (IC_50_: 3.8 µM), and curcumin (IC_50_: 1.6 µM). Resveratrol inhibited MERS‐CoV by downregulating the expression of the nucleocapsid [158] and resveratrol (from Sigma–Aldrich) further inhibited HCoV‐229E replication (EC_50_: 4.6 µM, CC_50_: 210 µM, SI: 45.65) [159]. Resveratrol is hampered by diminished water solubility and an extensive metabolism in the GI tract and liver [152]. Alternatives are actively sought to improve its efficacy in vivo. PEG‐stabilized resveratrol prohibited inflammatory storm and oxidative stress in MERS‐CoV with an EC_50_ of 0.0127 µg/mL and an SI of 724.4 with the nanocarrier compared to 7.3 without nanocarrier [28, 152].

Luteolin inhibits SARS‐CoV binding to Vero E6 host cells (EC_50_: 10.6 µM, CC_50_: 155 µM, SI: 14,6), while quercetin was not only less effective but also less toxic (EC_50_: 83.4 µM, CC_50_: 3320 µM) [160]. Hesperidin, EGCG, and kaempferol inhibited SARS‐CoV‐2 M^pro^ and spike protein in silico [161]. Luteolin, daidzein, apigenin, amentoflavone, epigallocatechin, and gallocatechin gallate also showed inhibition of M^pro^ [162].

In silico, catechins and curcumin interact with angiotensin‐converting enzyme 2 (ACE2), hampering the S protein binding to the receptor [163]. Resveratrol, together with low fat, had favorable effects on ACE2 expression [164].

Gossypol was characterized as a pan‐coronavirus‐inhibitor, for example, by targeting RdRps [165], and was particularly antiviral against SARS‐CoV‐2 with an EC_50_ of 0.76 µM, CC_50_ of 39.7 µM, and a therapeutic index (TI) of 52.07 [165].

## 5 Discussion

Polyphenols have gained medicinal attention as they exhibit antiinflammatory, antioxidant, and antimicrobial activities [166]. This review underscored the antiviral potential of polyphenols against enveloped viruses of clinical relevance. The most active polyphenols were the flavonoids, which could affect the infection cycles of arboviruses, coronaviruses, and hepadnaviruses. The molecules with the broader effects were EGCG (which affected virion structure, attachment, internalization, transcription, replication, and antiviral response) and reseveratrol (which hampers internalization, antiviral response, transcription, replication, and cell cycle modulation). The most sensitive viruses were the herpesviruses, which were susceptible to all classes of polyphenols, except for flavonoids.

The antiviral properties inherent in the polyphenols are based on several pathways. The most commonly affected step in viral infection is the replication phase due to interference of either DNA (herpesviruses, poxviruses, and hepadnaviruses) or RNA (arboviruses and FLUVs) polymerases. Virtually all polyphenols described herein were shown to interfere with this step, reflecting the complexity and susceptibility to disruption of this process. Equally affected was the attachment phase. This step was affected by fewer poylphenols than replication (chebulagic and chebulinic acids, EGCG, geraniin, luteolin, and psiloxylon) but involved several viruses (arboviruses, herpesviruses, coronaviruses, filoviruses, and FLUVs).

The second most common pathway affected by polyphenols (baicalin, caffeic acid, EGCG, gossypol, honokiol, luteolin, manassantin, PGG, piceatannol, quercetin, resveratrol, and wogonin) was transcription of viral genes. The lower susceptibility of viruses to transcriptional hindrance might be explained by the fact that this step relies on cellular enzymes more than the replicative step. Hindrance of transcription affected a wide range of viruses (herpesviruses, HBV, ZIKV, and FLUV). Since both replication and transcription are postinfection steps, it is feasible to assume that polyphenols can be used as therapeutic agents.

The therapeutic value of polyphenols can also be extended for specific viruses (herpesviruses, FLUVs, and coronaviruses) by the hindrance of steps such as nuclear export (resveratrol), translation (ginkgolic acid and pinocembrin), proteolysis (XN and punicalagin), modulation of inflammation (piceatanol, pyrogallol, resveratrol), and enhancement of antiviral response (TCQA). Since the latter two steps involve the modulation of the cellular oxidative stress, the polyphenols involved in these processes might have a particular clinical interest.

In addition, some polyphenols can act as prophylactic molecules by affecting the attachment (chebulagic acid, chebulinic acid, geraniin, luteolin, EGCG, and psiloxylon), internalization (EGCG, ginkgolic acid), and even viral structure (curcumin and EGCG), suggesting that these molecules can prevent the infection of selected viruses (arboviruses, FLUVs, and herpesviruses).

**FIGURE 4:**
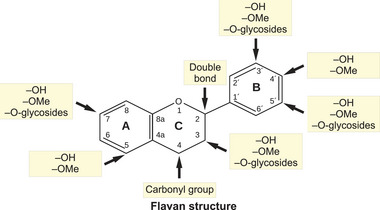
Biologically relevant residues of flavonoids. Basic structure of the flavan skeleton with numeration of the carbon atoms and identification of the two aromatic and the heterocyclic rings (A–C) with indication of the most relevant positions and their main substituents like hydroxyl groups (–OH), methoxy groups (–OMe), or –*O*‐glycosides, which are important for the antimicrobial activity of the flavonoids.

Despite the auspicious preclinical effects reported by in silico, in vitro, and in vivo experiments, further studies are required to discover the mechanisms of antiviral components in suppressing viral infections, like impaired entry mechanisms, eliciting a high immune response, and overcoming resistance to established drugs like boosting drug activities. There are specific positions within the flavan structure that are more relevant for determining the biological activities of flavonoids (Figure 4) [167]. Interestingly, it has been shown that the groups more involved in modulating the antioxidant activity of flavonoids also determine the biological activity of these molecules. The presence of hydroxyl or methoxy groups, double carbon bonds in the C‐ring, and glycosylation in the B‐ring have been associated with an enhancement of their antimicrobial properties [168]. In EGCG, the presence of 3‐galloyl and hydroxyl groups in position 5′ of the B‐ring has been associated with the antiviral activity of this molecule [169]. For the other polyphenols, the link between structure and antimicrobial function is still poorly understood.

Although natural compounds are expected to have good tolerability and a low rate of side effects, the low bioavailability and rapid degradation of many polyphenols like resveratrol in the body is one of a variety of causes that hamper the development of antiviral drugs from natural compounds. Strategies to enhance bioavailability could provide an attractive subject for future studies.

## 6 Conclusions

According to the literature gathered by the present review, polyphenols can affect the replication of enveloped human pathogenic viruses at several molecular points. In particular, EGCG and resveratrol had the broader effect on the viral infection, whereas herpesviruses were the viruses most affected by polyphenols. Numerous in silico, in vitro, and in vivo studies underline the great potential of various polyphenols for use in viral infections.

## Conflicts of Interest

The authors declare no conflicts of interest.

## Peer Review

The peer review history for this article is available at https://publons.com/publon/10.1002/mnfr.70042.

## Data Availability

Data sharing is not applicable to this article as no new data were created or analyzed in this study.
